# A new species of the genus *Blaptogonia* from the Himalayas with four DNA markers (Coleoptera, Tenebrionidae, Blaptini)

**DOI:** 10.3897/zookeys.773.24656

**Published:** 2018-07-09

**Authors:** Xiu-Min Li, Xing-Long Bai, Guo-Dong Ren

**Affiliations:** 1 The Key Laboratory of Zoological Systematics and Application, College of Life Sciences, Hebei University, Baoding, Hebei 071002, P.R. China

**Keywords:** biology, darkling beetles, DNA sequence, taxonomy, Tenebrioninae

## Abstract

A new species of the genus *Blaptogonia* Medvedev, 1998, *B. zhentanga* **sp. n.**, is described from the southern Himalayas of China. Two fragments of mitochondrial protein-coding genes (COI, Cytb), one fragment of mitochondrial ribosomal RNA gene (16S), and one fragment of nuclear rRNA gene (28SD2) of the new species were obtained. A key to the known species of the genus is presented.

## Introduction

The tenebrionid genus *Blaptogonia* Medvedev, 1998 belongs to the subtribe Gnaptorinina Medvedev, 2001 of tribe Blaptini Leach, 1815 within the subfamily Tenebrioninae Latreille, 1802. To date, only four species have been described worldwide and are known to occur only in the southern Himalayas at 3000–4000 meters (Medvedev 2004).

The first species, *Trigonoides costulata* Fairmaire, 1901, was described from Sikkim, India. It is evident from the text that *Trigonoides* used by Fairmaire (1901) is an incorrect spelling for *Tagonoides* Fairmaire, 1886. The second species, *Blaps subcarinata* Blair, 1927, was described based on the specimens of the third expedition to Mt. Everest from Tibet, China. Subsequently, a new genus, *Blaptyscelis*, was established by Koch (in Pierre, 1961), along with the combinations *Blaptyscelis costulata* Fairmaire, 1901 and *B. subcarinata* Blair, 1927. Koch (1965) redefined the diagnostic characters of this genus, which were accepted by Medvedev (1998, 2004). The third species, *Blaptyscelis zurstrasseni* Kaszab, 1977 was described from Nepal. A contribution to the knowledge of the tribe Blaptini was subsequently made by Medvedev (1998) who established the replacement name *Blaptogonia* since *Blaptyscelis* Koch was originally proposed without a type species designation. Thus, the combinations *Blaptogonia costulata* Fairmaire, 1901, *B. subcarinata* Blair, 1927, and *B. zurstrasseni* Kaszab, 1977 were established, and *Tagonoides costulata* Fairmaire, 1901 was designated as the type species.

At the beginning of the 21st century, *Blaptogonia yini* Ren, Wang & Yu, 2000 was described from Tibet and placed in *Blaptogonia* because of the distinct elytral carinae, one of the typical characters of the genus, but was moved recently to the genus *Blaps* Fabricius, 1775 (Ren et al. 2016) on the basis of additional materials and structures of male genitalia. The fourth species, *Blaptogonia tshernjachovskii* Medvedev, 2004, was described from Nepal based on two female specimens. Medvedev (2004) also characterized the structures of the apical part of the male abdomen, as well as the ovipositor and genital tubes of the female, as diagnostic characters of the genus *Blaptogonia*.

In the present study, a new species of the tribe Blaptini, collected from southern Qomolangma Nature Reserve of Tibet, is described. In addition, two fragments of mitochondrial protein-coding genes (COI, Cytb), one fragment of mitochondrial ribosomal RNA gene (16S), and one fragment of nuclear rRNA gene (28SD2) of the new species were sequenced and uploaded to GenBank.

## Materials and methods

### Morphology

All specimens examined in this study were deposited in the Museum of Hebei University (**MHBU**), Baoding, China. Photographs of morphological structures were taken using a Leica M205A stereomicroscope with a Leica DFC550 camera and Leica application suite 4.6. The habitus photos were taken using a Canon EOS 5D Mark III camera connected to a Canon Macro lens MP-E 65 mm.

### DNA extraction, PCR amplification, and sequencing

Total DNA was extracted from leg muscle tissue of a single adult specimen using EZNA® Insect DNA Kit (Omega Bio-tek, USA). Total DNA extract was stored at -20 °C. Two fragments of mitochondrial protein-coding genes (COI, Cytb), one fragment of mitochondrial ribosomal RNA gene (16S), and one fragment of nuclear rRNA gene (28SD2) were amplified using the primers of Table 1. The profile of the PCR amplification consisted of an initial denaturation step at 94 °C for 4 min, 35 cycles of denaturation at 94 °C for 1 min, annealing at 50–58 °C for 1 min, and extension at 72 °C for 1 min, and a final 8 min extension step at 72 °C. The PCR products were subsequently checked by 1% agarose gel electrophoresis and sequencing was performed at GENEWIZ Biotech Co., Ltd. (Suzhou, China) using the same primers as in the PCR.

**Table 1.** Primer sequences for PCR.

Locus	Primer (Forward /Reverse/ Internal)	Sequence (forward and reverse) 5’→3’	References
COI	F 2183	CAACATTTATTTTGATTTTTTGG	Folmer et al. 1994
	R 3014	TCCAATGCACTAATCTGCCATATTA	
Cytb	F revcb2h	TGAGGACAAATATCATTTTGAGGW	Simmons and Weller 2001
	R rebcbj	TCAGGTCGAGCTCCAATTCATGT	
16S	F 13398	CGCCTGTTTATCAAAAACAT	Simon et al. 1994
	R 12887	CCGGTCTGAACTCAGATCAT	
28SD2	F 3665	AGAGAGAGTTCAAGAGTACGTG	Belshaw and Quicke 1997
	R 4068	TTGGTCCGTGTTTCAAGACGGG	

## Results

### Key to known species of the genus *Blaptogonia*

**Table:** 

1	Surface of elytra between carinae without subcarinae (i.e. secondary carinae)	**2**
–	Surface of elytra between carinae with subcarinae	**3**
2	Both carinae on each elytron evanescent in the basal half and 2^nd^ carina not reaching humeral carina at the apex in male and female	***B. zurstrasseni* Kaszab, 1977**
–	Both carinae on each elytron reaching the elytral base and fused with humeral carina at the apex in female	***B. tshernjachovskii* Medvedev, 2004**
3	Elytral surface between carinae and subcarinae with fine granules; carinae higher than subcarinae; paramere arcuately concave, narrowing from basal 1/5 to apex	***B. zhentanga* sp. n.**
–	Elytral surface between carinae and subcarinae without granules; carinae as high as subcarinae; paramere almost straight, Parameres narrowing from base to apex	**4**
4	Body and legs black; male elytra more oval, with a row of small punctures between 4^th^ and 5^th^ carinae, and between 5^th^ and humeral carinae	***B. costulata* Fairmaire, 1901**
–	Head and elytra dark brown, pronotum and legs reddish brown; male elytra narrow, more parallel sided, without rows of punctures between carinae and subcarinae	***B. subcarinata* Blair, 1927**


#### Genus Blaptogonia

Taxon classificationAnimaliaColeopteraTenebrionidae

Medvedev, 1998

Blaptogonia Medvedev, 1998: 186; 2001: 95; 2004: 89; Löbl et al. 2008: 231; Ren et al. 2016: 332.

##### Type species.

*Tagonoides costulata* Fairmaire, 1901.

#### Blaptogonia costulata

Taxon classificationAnimaliaColeopteraTenebrionidae

(Fairmaire, 1901)

Trigonoides costulata Fairmaire, 1901: 267.
Blaptyscelis costulata : Koch in Pierre 1961: 213.
Blaptogonia costulata : Medvedev 1998: 186; 2001: 95; 2004: 89; Löbl et al. 2008: 231.

##### Distribution.

India: Sikkim.

#### Blaptogonia subcarinata

Taxon classificationAnimaliaColeopteraTenebrionidae

(Blair, 1927)

Blaps subcarinata Blair, 1927: 243.
Blaptyscelis subcarinata : Koch in Pierre 1961: 213.
Blaptogonia subcarinata : Medvedev 1998: 186; 2001: 95; 2004: 89; Löbl et al. 2008: 231; Ren et al. 2016: 332.

##### Distribution.

China: Tibet (Yadong, also called Chomo).

#### Blaptogonia tshernjachovskii

Taxon classificationAnimaliaColeopteraTenebrionidae

Medvedev, 2004

Blaptogonia tshernjachovskii Medvedev, 2004: 177; Löbl et al. 2008: 231.

##### Distribution.

Nepal.

#### Blaptogonia zhentanga sp. n.

Taxon classificationAnimaliaColeopteraTenebrionidae

http://zoobank.org/4B416851-9F94-4EEB-B369-424908D08D54

Figs 1 , 2 , 3–4

##### Type material.

**Holotype**: male (MHBU) (Fig. 3), **CHINA**: Xizang, Dinggyê County, Zhêntang Town, Power Station, 27°55.069'N, 87°28.171'E, Alt. 3418 m, 4.VIII.2014, Guo-Dong Ren, Xing-Long Bai & Jun-Sheng Shan leg. **Paratypes**: 54 males, 28 females (MHBU), same data as the holotype.

##### Diagnosis.

This new species is closely related to *Blaptogonia subcarinata* Blair, 1927 but can be distinguished from the latter by the following character states: (1) elytral surface with fine granules and irregular and shallow fine punctures, whereas *subcarinata* has no granules and rows of punctures between carinae and subcarinae; (2) elytral carinae more elevated than the subcarinae, whereas the subcarinae are as high as the carinae in *subcarinata*; (3) parameres arcuately concave and narrowing from basal 1/5 to apex, whereas the parameres are nearly straight in *subcarinata* and narrowing from base to apex.

##### Etymology.

Named after the type locality, Zhêntang.

##### Description.

Head, palps, antennae, carinae, subcarinae, humeral carina, abdomen, tibiae, and tarsus black. Pronotum, elytra, femora, apical spurs, and claws reddish brown to brown. Shiny dorsally and ventrally, with sparse and short pubescence at apex on elytra.

**Figure 1.**
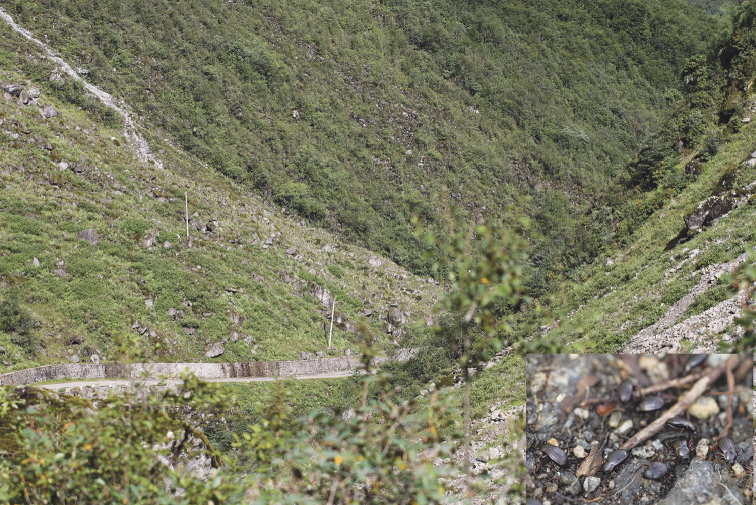
Habitat and population of *Blaptogonia zhentanga* sp. n.

**Figure 2.**
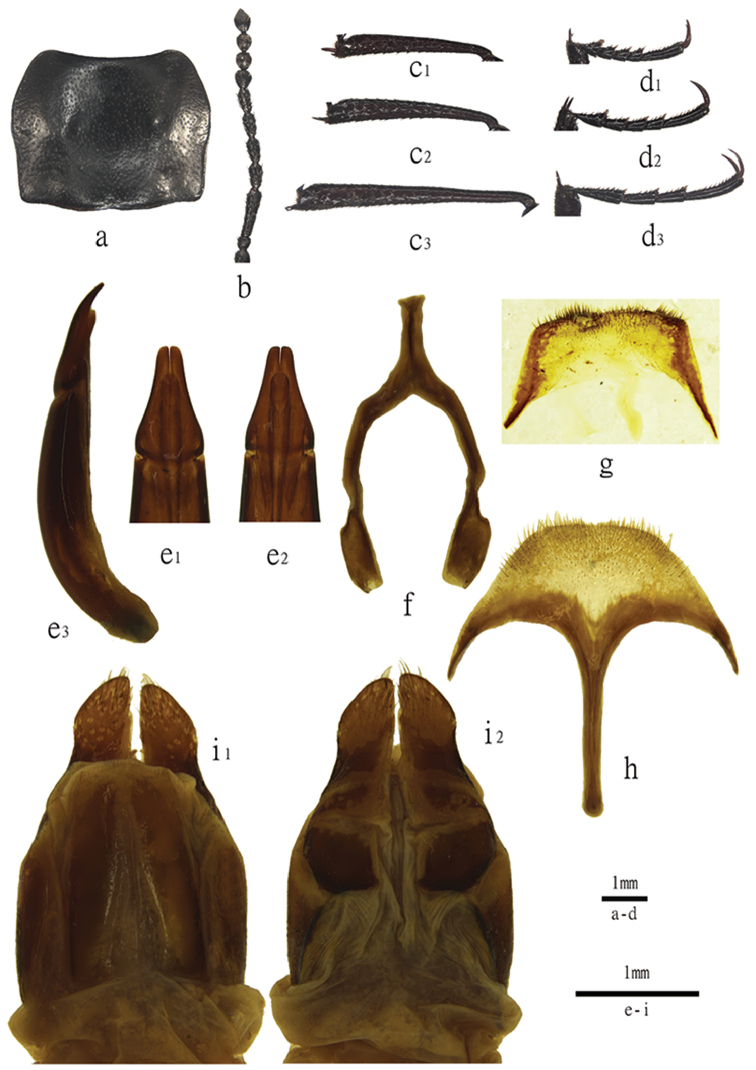
Characters of *Blaptogonia zhentanga* sp. n. **a–g** male: **a** pronotum **b** antenna **c_1_–c**_3_ pro-, meso, metatibia **d_1_–d_3_** pro-, meso-, metatarsus **e_1_–e_3_** aedeagus in dorsal, ventral, and lateral view **f** *spiculum astrale* **g**. abdominal sternite VIII **h–i** female: **h** *Speculum ventrale* in ventral view **i_1_–i_2_** ovipositor in dorsal and ventral view.

**Figures 3–4.**
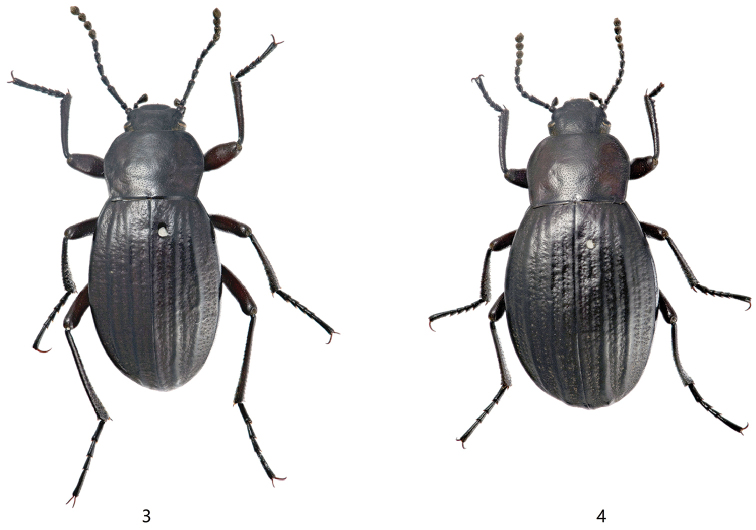
Habitus of *Blaptogonia zhentanga* sp. n. **3**.male, holotype, 12.2 mm **4** female, paratype, 13.5 mm.

**Male** (Figs 2a–g, 3). Labrum with sparse punctures and pale yellow setae. Anterior margin of clypeus straight, tilted at sides, surface with sparse fine punctures; frontoclypeal suture shallow. Anterior gena slightly extended before eyes, outer margins straight and converging toward base of clypeus, surface with moderately dense fine punctures; emargination of outer margins of head above antennal base widely obtuse-angular. Eyes transverse, weakly projecting and clearly wider than anterior gena. Posterior gena densely hairy, outer margins arcuately converging to neck. Surface of head with moderately dense punctures. Antennae (Fig. 1b) moderately long, with apical segment reaching beyond pronotal base, length (width) ratio of antennomeres 2 to 11 are 1.0(0.9): 1.0(0.3): 1.0(0.5): 1.0(0.5): 1.0(0.5): 1.0(0.5): 1.0(1.0): 1.0(0.8): 1.0(0.9): 1.0(0.7).

*Pronotum* (Fig. 2a) transverse, widest before middle, 1.3–1.5 times as wide as long, 1.6–1.8 times as wide as head. Ratio of pronotal width at anterior margin to its maximum and base 0.6: 1.0: 0.9. Anterior margin arcuate, bordered laterally by bead along entire length; posterior margin weakly arcuate, without bead. Anterior angles obtuse, rounded apically; posterior angles obtuse. Pronotal surface between lateral margins weakly convex, with moderately dense and shallow fine punctures on disc. Propleura with fine shallow wrinkles. Prosternal process with dense pale hairs.

*Elytra* elongate-oval, 1.4–1.6 times as long as wide, and 1.2–1.4 times as wide as pronotum. Each elytron between suture and humeral carina with two distinct carinae. In addition, surface of elytra between suture and 1^st^ carina, 1^st^ and 2^nd^ carina, 2^nd^ and humeral carina with lower subcarinae; subcarinae between 2^nd^ and humeral carina indistinct. The humeral carina, subcarinae and carinae reaching base of elytra; 1^st^ carina fused with humeral carina at apex, 2^nd^ carina and subcarinae indistinct at apex. Surface of suture, subcarinae, carinae, humeral carina, between suture and subcarinae, between subcarinae and carinae, between subcarinae and humeral carina with irregular sparse and shallow fine punctures, sparse fine granules, and shallow wrinkles; punctures and wrinkles indistinct at apex. Epipleura not reaching suture of elytral angle, outer margin visible in dorsal view only at humeri, sometimes at apices. Visible abdominal sternites covered with short pale recumbent setae, sparse and fine shallow punctures, and fine granules; 1^st^ to 3^rd^ ventrites with fine longitudinal wrinkles, 2^nd^ ventrite flattened in the middle, 4^th^ ventrite shallowly depressed at sides.

*Legs* (Fig. 2c, d) rather slender. Protibia (Fig. 2c_1_) straight, inner apical spur longer than outer one; mesotibia (Fig. 2c_2_) nearly straight; metatibia (Fig. 2c_3_) straight, inner apical spur equal to or slightly longer than outer one. Ventral surface of protarsomere I (Fig. 2d_1_) with hairy brush, mesotarsomere I (Fig. 2d_2_) with hairy tuft. Length (width) ratio of pro-, meso- and metafemora 7.4(1.0): 2.5(1.0): 3.0(1.0), that of corresponding tibiae 3.4(1.0): 3.5(1.0): 4.3(1.0); that of metatarsomeres (Fig. 1d_3_) 12.7(1.0): 7.3(1.0): 6.5(1.0): 13.8(1.0). Claws slender, longer than half the length of apical tarsomere.

*Aedeagus* (Fig. 2e–g): length 2.9 mm, width 0.8 mm. Parameres 0.9 mm long and 0.6 mm wide, arcuately concave, narrowing from basal 1/5 to apex (Fig. 2e_1_, e_2_), and curved to ventral side in lateral view (Fig. 2e_3_).

**Female** (Figs 2h–i, 4). Body wider than in male. Antennae reaching pronotal base. Pronotum 1.4–1.5 times as wide as long, 1.6–1.8 times as wide as head. Elytra 1.3–1.5 times as long as wide, and 1.4–1.6 times as wide as pronotum. Second visible sternite not flattened in middle. Apical spur slender, sharp at apex; inner apical spur of protibia slightly longer than outer one. Ventral surface of tarsus without hairy tuft. *Spiculum ventrale* as in Fig. 1h. Ovipositor as in Fig. 1i.

##### Measurements.

Body length: male 11.9–13.8 mm, female 13.1–14.9 mm; width: male 5.3–6.0 mm, female 7.0–7.4 mm.

##### Distribution.

China: Xizang.

##### Molecular characters.

Two fragments of mitochondrial protein-coding genes (COI, Cytb), one fragment of mitochondrial ribosomal RNA gene (16S), and one fragment of nuclear rRNA gene (28SD2) are deposited in GenBank with the accession numbers MG946798, MG946797, MG946800, and MG946799, respectively, based on one male, China, Xizang, Dinggyê, Zhêntang, 4 August 2014, coll. Guo-dong Ren, Xing-long Bai & Jun-sheng Shan. The sequence is presented in Table 2.

**Table 2.** Sequence of *Blaptogonia zhentanga* sp. n.

**GenBank accession No.**	**Locus**	**Sequence**
MG946798	COI	TGCCATATTAGAATGATGACAGTATAGGGAGTTCAGAATATCTGTGTTCAGCAGGGGGAGTATTTTGTAATCATTCAATAGAGGAGGTTATATTAAGCGAGGTTAAGGATTTTCGTGATGAAGAAAATCTTTCTCATATAATGAAAATTAGGAATAATACTCCTACTAAAGATATTAGAGACCCAATTGAGGAAATAATATTTCATAGGGTGTAGGCATCAGGGTAATCTGAGTATCGTCGGGGTATTCCTCTTAATCCGAGAAAGTGTTGAGGAAAGAAGGTAAGGTTTACGCCCACGAATATTACAAAAAATTGAATTTTACACAGTTTTGCCCTTAAGGATAAACCTGTGAATAAAGGGAATCAATGTACTAATCCTCCTAGAATTGCAAATACAGCTCCTATAGATAATACGTAATGGAAATGGGCTACTACATAATAGGTATCATGTAATATAATATCAATGGAAGAGTTAGCTAGAATTACTCCTGTTAATCCTCCTACTGTAAATAAAAATACGAATCCTAATGCTCATAATATTGAGGGACTATAATTTAGTTGTGTTCCGTGGAGAGTGGCTAATCATCTGAAAATTTTAATTCCAGTAGGAACTGCAATAATTATTGTTGCTGAAGTGAAATATGCTCGAGTATCTACGTCTATTCCTACTGTAAATATGTGATGGGCTCATACCACAAATCCTAATAATCCAATTGCTATTATAGCATAAATTATTCCCAATGTTCCAAAGGCCTCTTTTTTACCTCTTTCTTGTCT
MG946797	Cytb	ATCATTTTGAGGTGCTACTGTAATTACAAACTTACTTTCAGCAATTCCATACCTAGGATCAACTATTGTACAATGATTATGAGGAGGGTTTGCAGTAGACAATGCAACACTTACTCGATTTTTTGCATTCCATTTCCTTCTTCCATTTATTGTAACAGCAATAGTTATAATCCATTTACTGTTTCCCACCAAACAGGATCTAATAATCCCCTAGGATTAAACAGTAATATTGACAAAATTCCATTTCACCCATACTTTTCCTTTAAAGACATTATAAGATTGATTATTACAATTATAGCTCTTGTAATACTATCTATTAATAGACCCTATCTACTAGGAGATCCAGACAACTTTACACCCGCAAACCCTCTATCAACCCCAATTCATATCCAGCCAGAATGATATTTCCTATTTGCTTATGCAATCTTACGTTCAATCCCTAATAAATTAGGAGGAGTAATTGCACTAGCAATATCAATTGCAATCCTTTATATTTTACCTCTATCTAATAAAAAAAAATTTGCAAGAAACTCATTTTACCCTATAAATAAAATCCTATTCTGAATTATATTAGTTACAGTGATTCTATTAACATGAATTGG
MG946799	28SD2	TTCAAGACGGGTCCTGAAAGTACCCAAAGCTATAGCGTCCGCAGATCGGCGTTTCAACGAGGTCCTGTTCGAGAACACCTCGGCCAACAGTCGCCCGGGGACGGGACCGGCACCAGGTCCGATCACCGTCGCGAGAAGCGCGCTTGCCGAAGGTCGAACGCTAACTGAATAGCGGCCCCGCGCCATCTGTATATCGTCGAGCGAGCCGACCGGGAAACACCGAGGGTTCGTCACGAACGCCGAAACGTCCGGACGAACTCCACCTCGGGCCTTAGGCCGACACCCAACGAATCGCGACGTCCTACAGGGGGAGAAGTGCACGCGTTCGACCGCAGTTCGGAGGACGAAAGGCGCGGACGACGCGTACGCCGTCCGTCGCACGACCATCCAGCGCCACGATCGAGACACGCTGAATCTCCCCTTTCGACCTTTCGGGTTTCTCAGGTTTACCCCTGAACGGTTTCACGTACTCTT
MG946800	16S	AAAAACATGTCTTTTTGTTTTATGATTTAAAGTCTGGCCTGCCCAATGATTAATTTTAAATGGCTGCAGTATTTTGACTGTACAAAGGTAGCATAATCATTAGTTTCTTAATTAGAAGCTGGAATGAATGGTTTGATGAAAAATTTACTGTCTCAATTCAATTGTTTTAGAATTTTATTTTTAAGTGAAAAAGCTTAAATTTTTTAGAAAGACGAGAAGACCCTATAGAGTTTTATATGTTTTTTATTATTTATTATATGGTTATAATATTTTTAATTTTAAAATTTATTTTGTTGGGGTGATGTGAAAATTTAAATAACTTTTCTTAATTTTAACACTAATTAGTGATTAAATGATCCTTTTTAGGATTAAAAGATTAAATTACCTTAGGGATAACAGCGTAATTTTTTTTGAAAGTTCTTATTGATAAAAAAGTTTGCGACCTCGATGTTGGATTAAAATTTATTTTTGGTGTAGAAGCTGGAAAATTTGGGTCTGTTCGACCCTTAAAATTTTACATGATCTG

##### Biology.

Adults of the new species were found beneath stones in the shrubbery (Fig. 1), usually with more than ten individuals per stone. When threatened, they released quite foul smells and irritating liquids from their abdominal defensive glands. The smell persisted for a few days in the laboratory.

#### Blaptogonia zurstrasseni

Taxon classificationAnimaliaColeopteraTenebrionidae

(Kaszab, 1977)

Blaptyscelis zurstrasseni Kaszab, 1977: 246.
Blaptogonia zurstrasseni : Medvedev,1998: 186; 2001: 95; 2004: 89; Löbl et al. 2008: 231.

##### Distribution.

Nepal.

## Supplementary Material

XML Treatment for Blaptogonia

XML Treatment for Blaptogonia costulata

XML Treatment for Blaptogonia subcarinata

XML Treatment for Blaptogonia tshernjachovskii

XML Treatment for Blaptogonia zhentanga

XML Treatment for Blaptogonia zurstrasseni
